# Immunomodulatory sphingosine-1-phosphates as plasma biomarkers of Alzheimer’s disease and vascular cognitive impairment

**DOI:** 10.1186/s13195-020-00694-3

**Published:** 2020-09-30

**Authors:** Xin Ying Chua, Yuek Ling Chai, Wee Siong Chew, Joyce R. Chong, Hui Li Ang, Ping Xiang, Kaddy Camara, Amy R. Howell, Federico Torta, Markus R. Wenk, Saima Hilal, Narayanaswamy Venketasubramanian, Christopher P. Chen, Deron R. Herr, Mitchell K. P. Lai

**Affiliations:** 1 Department of Pharmacology, Yong Loo Lin School of Medicine, National University of Singapore, Kent Ridge, 117597 Singapore; 2 Memory, Aging and Cognition Centre, National University Health Systems, Kent Ridge, Singapore; 3 Cancer Science Institute, National University of Singapore, Kent Ridge, Singapore; 4 Department of Chemistry, University of Connecticut, Storrs, CT USA; 5 Singapore Lipidomics Incubator (SLING), Department of Biochemistry, Yong Loo Lin School of Medicine, National University of Singapore, Kent Ridge, Singapore; 6 Saw Swee Hock School of Public Health, National University of Singapore, Kent Ridge, Singapore; 7 Raffles Neuroscience Centre, Raffles Hospital, Singapore, Singapore; 8 Department of Biology, San Diego State University, San Diego, CA USA

**Keywords:** Alzheimer’s disease, Biomarkers, Immunomodulation, Neuroinflammation, Sphingosine-1-phosphate, Vascular cognitive impairment

## Abstract

**Background:**

There has been ongoing research impetus to uncover novel blood-based diagnostic and prognostic biomarkers for Alzheimer’s disease (AD), vascular dementia (VaD), and related cerebrovascular disease (CEVD)-associated conditions within the spectrum of vascular cognitive impairment (VCI). Sphingosine-1-phosphates (S1Ps) are signaling lipids which act on the S1PR family of cognate G-protein-coupled receptors and have been shown to modulate neuroinflammation, a process known to be involved in both neurodegenerative and cerebrovascular diseases. However, the status of peripheral S1P in AD and VCI is at present unclear.

**Methods:**

We obtained baseline bloods from individuals recruited into an ongoing longitudinal cohort study who had normal cognition (*N* = 80); cognitive impairment, no dementia (*N* = 160); AD (*N* = 113); or VaD (*N* = 31), along with neuroimaging assessments of cerebrovascular diseases. Plasma samples were processed for the measurements of major S1P species: d16:1, d17:1, d18:0, and d18:1, along with pro-inflammatory cytokines interleukin (IL)-6, IL-8, and tumor necrosis factor (TNF). Furthermore, in vitro effects of S1Ps on cytokine expression were also studied in an astrocytoma cell line and in rodent primary astrocytes.

**Results:**

Of the S1Ps species measured, only d16:1 S1P was significantly reduced in the plasma of VaD, but not AD, patients, while the d18:1 to d16:1 ratios were increased in all cognitive subgroups (CIND, AD, and VaD). Furthermore, d18:1 to d16:1 ratios correlated with levels of IL-6, IL-8, and TNF. In both primary astrocytes and an astroglial cell line, treatment with d16:1 or d18:1 S1P resulted in the upregulation of mRNA transcripts of pro-inflammatory cytokines, with d18:1 showing a stronger effect than d16:1. Interestingly, co-treatment assays showed that the addition of d16:1 reduced the extent of d18:1-mediated gene expression, indicating that d16:1 may function to “fine-tune” the pro-inflammatory effects of d18:1.

**Conclusion:**

Taken together, our data suggest that plasma d16:1 S1P may be useful as a diagnostic marker for VCI, while the d18:1 to d16:1 S1P ratio is an index of dysregulated S1P-mediated immunomodulation leading to chronic inflammation-associated neurodegeneration and cerebrovascular damage.

## Introduction

Alzheimer’s disease (AD) and vascular dementia (VaD) are the two major causes of cognitive impairment in the elderly and represent a major healthcare burden on both developed and developing countries [1, 2]. AD is characterized by neurodegeneration associated with abnormally aggregated β-amyloid and hyper-phosphorylated tau proteins, while VaD, falling under the spectrum of vascular cognitive impairment (VCI) [3, 4], predominantly manifests cerebrovascular disease (CEVD) involving ischemic or atherosclerotic lesions of small vessels in the brain parenchyma and subarachnoid spaces. Nevertheless, cumulative data have suggested that (i) AD and CEVD pathologies frequently coexist [5–8]; (ii) AD and CEVD share major pathogenic factors, including neuroinflammation and oxidative stress [9–11]; and (iii) CEVD interacts in an additive or synergistic manner with AD in contributing to dementia severity [7, 12–15]. These observations point to the importance of assessing the co-occurence, type, and severity of CEVD in longitudinal studies of AD progression, particularly those aiming to uncover prognostic biomarkers. Furthermore, given the extended prodromal stages where potential disease-modifying therapies may more likely be efficacious [16], especially for certain forms of small vessel CEVD [14, 17], the availability of reliable, easily measurable diagnostic biomarkers is essential for optimal clinical management and will also help in the advancement and assessment of new therapeutic strategies for AD and VCI. To this end, the development of blood-based biomarkers has been ongoing, with novel markers for a wide range of processes being evaluated [3, 18–21]. However, few studies have focused on uncovering markers that may discriminate between AD- and CEVD-driven disease burden in patients where these processes coexist.

Sphingosine-1-phosphates (S1Ps) are a group of structurally simple, mono-acylated bioactive lipids with a well-defined metabolic pathway [22]. They are highly pleiotropic signaling molecules that mediate biological effects primarily by activating a family of five G protein-coupled receptors (S1PR_1_–S1PR_5_, [23]). In mammals, the 18-carbon mono-unsaturated S1P (d18:1) is the most abundant form, making up approximately 80% of total S1P in the plasma [24]. However, other structural variants that vary in chain length and degree of saturation are known to exist in physiologically relevant concentrations that exceed the EC_50_ of cognate S1P receptors [24, 25]. Studies into the biological roles of S1P have been largely limited to d18:1 S1P. As a result, the relevance of less abundant S1P species is currently unclear.

S1P-mediated receptor signaling has been shown to regulate a variety of processes in central nervous system cells, such as differentiation, survival and excitability of neurons, activation of astrocyte-mediated neuroinflammation, and processing of amyloid precursor protein [26]. Therefore, it is plausible that S1P signaling is altered in individuals suffering from dementia, which may in turn result in measurable changes in circulating S1P levels and may serve as biomarkers for AD- or CeVD-driven processes.

In this study, we determined the plasma concentrations of the four most abundant S1P species in a community-based cohort of dementia patients in Singapore. We evaluated these lipids as potential biomarkers for AD and VCI and used cell-based assays to examine postulated pathophysiological mechanisms underlying the clinical findings.

## Methods

### Study cohort, medical and cognitive assessments

Institutional Review Board approval for the study was obtained from the Singapore National Healthcare Group Domain-Specific Review Board (reference 2010/00017; study protocol DEM4233), and written informed consent had been obtained from participants or their next-of-kin before study recruitment and blood collection procedures. The selection and assessment of the cohort for this case-control study, which represents baseline measurements of an ongoing longitudinal study, have been previously described [27–29]. Briefly, patients with subjective complaints of memory loss were recruited from memory clinics at Singapore’s National University Hospital and St Luke’s Hospital sites. All subjects underwent clinical, physical, and neuropsychological assessments and neuroimaging at the National University of Singapore. Relevant demographic and medical information, including vascular risk factors (see the “Covariates” section) and exclusion factors such as previous head trauma, thyroid disease, non-AD neurodegenerative conditions (e.g., Parkinson’s disease), and psychiatric illnesses, were collected by administering a detailed questionnaire and reviewing of medical records. Furthermore, subjects were administered a comprehensive neuropsychological test battery consisting of several domains, namely, executive function, attention, language, visuomotor speed, visuoconstruction, verbal memory, and visual memory [27], along with standard cognitive assessments (Mini-Mental State Examination [30] and Montreal Cognitive Assessment [31]). Diagnoses of cognitive impairment and dementia were made at regular consensus meetings of study clinicians and neuropsychologists, where cognitive impairment, no dementia (CIND) cases were defined by people who did not meet the Diagnostic and Statistical Manual Fourth Edition (DSM-IV) diagnostic criteria for dementia [32] but showed impairment in one or more domains of the neuropsychological battery, as defined by education-adjusted scores ≥ 1.5 standard deviations below normal established means for at least half of the tests for that domain. AD cases were diagnosed using the National Institute of Neurological and Communicative Disorders and Stroke and the Alzheimer’s disease and Related Disorders Association (NINCDS-ADRDA) criteria [33], while vascular dementia (VaD) was diagnosed using the National Institute of Neurological Disorders and Stroke-Association Internationale pour la Recherché et l’ Enseignement en Neuroscience (NINDS-AIREN) criteria [34]. Non-cognitively impaired (NCI) controls were defined as those with subjective memory complaints, but who were found to be cognitively normal after undergoing objective neuropsychological assessments.

### Covariates

In addition to demographic information, medical histories of vascular risk factors such as hypertension, hyperlipidemia, diabetes, smoking, and cardiovascular disease were collected and classified as absent or present. Hypertension, hyperlipidemia, and diabetes mellitus were also determined by recorded use of antihypertensive, antihyperlipidemic, and anti-diabetic medications, respectively. Cardiovascular disease was determined by previous history of atrial fibrillation, congestive heart failure, and/or myocardial infarction. Apolipoprotein E (APOE) genotyping was performed as previously described [35] for the determination of APOE ε4 carrier status, defined by the presence of at least one APOE ε4 allele.

### Blood processing

Non-fasting blood was collected via venipuncture from study participants into both serum-separating tubes (SST) and ethylenediaminetetraacetic acid (EDTA)-containing tubes, followed by centrifugation at 2000*g* for 10 min at 4 °C. Serum (from SST tubes) or plasma (from EDTA tubes) was extracted and stored at − 80 °C until use.

### Liquid chromatography-tandem mass spectrometry (LC-MS/MS) measurements of plasma sphingolipids

All chemicals were purchased from Sigma-Aldrich (St Louis, MO, USA) and of reagent grade unless otherwise specified. d16:1 S1P was custom-synthesized as described in the Supplementary Information. An internal standard solution (ISTD) consisting of 20 ng/mL ^13^C_2_D_2_–d18:1 S1P in methanol was prepared. Ten microliters of each plasma sample was used for S1P extraction. Plasma samples were mixed with 100 μL of ISTD and sonicated for 30 min at 23 °C. Samples were then centrifuged at 16,000*g* for 10 min at 23 °C, after which the supernatant was aliquoted and vacuum-dried (Savant SpeedVac® vacuum concentrator, Thermo Fisher Scientific, Waltham, MA, USA). Dried lipid extracts were resuspended in 100 μL methanol and sonicated for 10 min at 23 °C. Derivatization of S1P was then carried out by adding 10 μL of TMS-diazomethane and shaking on thermomixer at 700 rpm for 20 min at 23 °C. After 20 min, 1 μL of 100% acetic acid was added to stop the derivatization reaction. Samples were vortexed, then centrifuged at 16,000*g* for 10 min to remove potential debris before transferring into a 96-well plate. Plates were stored at 4 °C until LC-MS/MS analyses using an Agilent 1290 UPLC system connected to an Agilent 6495 Triple Quadrupole mass spectrometer operated in positive ion MRM mode (Agilent Technologies, Santa Clara, CA, USA). The column utilized was an Acquity® hydrophilic interaction chromatography (HILIC) column (100 × 2.1 mm, 1.7 μm particle size) (Waters Corporation, Milford, MA, USA). MS source parameters used gas temperature of 200 °C with gas flow of 12 L/min and nebulizer at 25 psi. Sheath gas temperature of 400 °C with gas flow of 12 L/min. Solvents used for the HILIC are 50% acetonitrile in water containing 25 mM ammonium formate pH 4.7 (solvent A) and 95% acetonitrile in water containing 25 mM ammonium formate pH 4.7 (solvent B). Analytes were eluted with the following gradient: 0.1% solvent A and 99.9% solvent B from 0 to 5 min, 60% solvent A and 40% solvent B from 5 to 5.5 min, 90% solvent A and 10% solvent B from 5.5 to 6.6 min, 0.1% solvent A, and 99.9% solvent B from 6.6 to 9 min with a constant flow rate of 0.4 mL/min. After collision-induced dissociation of the S1P precursors, two productions were produced and monitored: m/z 60 was used as a “quantifier” and m/z 113 was used as the “qualifier.” These two ions were present after fragmentation of all S1P molecular species. The ion with m/z 60 represents the trimethylated amine fragment while the ion with m/z 113 represents the mono-methylated phosphate as previously described [24]. Quantification of the four S1P isoforms (Table 1) was performed based on the internal standard method, calibrating peak areas of the sample to the ISTD. Data were extracted and analyzed using Agilent MassHunter Qualitative and Quantitative software (Santa Clara, CA, USA). Principle Components Analysis (PCA) was carried out to ensure the results’ quality and validity.

**Table 1:** Nomenclature and probable molecular structures of S1P isoforms quantified in this study

Name	Nomenclature	Formula	Molecular structure
d16:1 S1P	Hexadecasphing-4-enine-1-phosphate	C_16_H_34_NO_5_P	
d17:1 S1P	Heptadecasphing-4-enine-1-phosphate	C_17_H_36_NO_5_P	
d18:0 S1P	Sphinganine-1-phosphate	C_18_H_40_NO_5_P	
d18:1 S1P	Sphing-4-enine-1-phosphate	C_18_H_38_NO_5_P	

### Measurements of peripheral cytokines

Measurements of acute inflammatory cytokines in serum are interleukin (IL)-6, IL-8, and tumor necrosis factor (TNF) by multiplex xMAP®-based Luminex immunoassays (MILLIPLEX, catalog number HADK2MAG-61 K, Merck Millipore, Billerica, MA, USA) which has been previously described [29]. Briefly, serum was incubated overnight with a mixture of fluorescent-coded magnetic beads coated with a specific capture antibody against each cytokine. This was followed by the addition of biotinylated antibody and streptavidin-phycoerythrin conjugates. Median fluorescent intensities (MFIs) of each cytokine were measured on a Luminex 200™ machine with xPONENT software (Luminex Corporation, Austin, TX, USA), and standard curves were fitted to a 5-parameter logistic model ranging from 0.96 to 15,000 pg/mL for IL-6 and 0.64 to 10,000 pg/mL for both IL-8 and TNF.

### Cell culture

U-373 MG cells (#08061901 Sigma-Aldrich, St Louis, MO, USA) and HEK293 cells (ATCC #CRL-1573) were maintained as monolayer cultures on tissue culture dishes at 37 °C, 5% CO_2_, and 100% humidity in Dulbecco’s modified Eagle’s medium (DMEM) supplemented with 10% heat-inactivated fetal bovine serum (FBS) and antibiotics. For S1P treatments, cells were grown to ~ 80% confluence, incubated for 4 h in serum-free media, and then treated with the indicated ligand(s) or vehicle for 2 h prior to collection of lysates.

Primary astrocytes were isolated from C57BL/6NTac mouse pups (from InVivos, Singapore; IACUC protocol approval #R14-0316, National University of Singapore) at postnatal day 0–3 with a modification of Schildge’s method [36]. Briefly, the cerebral hemispheres were collected and meninges were carefully removed. Cortical tissues were minced and then incubated in 1× Hank’s Balanced Salt Solution (HBSS) with 0.01% trypsin and 10 μg/mL DNase for 15 min at 37 °C. Dissociated cells were then passed through a 40-μm nylon cell strainer and placed in a poly-D-lysine-coated T75 flask with DMEM containing 10% FBS and 1% penicillin/streptomycin. The medium was changed every 2 to 3 days until mixed glial cells become confluent. Microglia and oligodendrocytes were then removed by shaking at 220 rpm for 18–20 h, and the remaining confluent astrocytes were attached. Purity of astrocytes was determined by glial fibrillary acidic protein (GFAP) immunostaining showing > 95% positive labeling (data not shown). Astrocytes were used from passage 1–3 for treatment with ligand(s) as described above for U-373MG cells. All cell culture experiments were independently performed 3–4 times.

### Quantitative reverse transcription PCR (RT-PCR)

mRNA quantification was performed essentially as described [37], with reagents and kits from Thermo Fisher Scientific (Waltham, MA, USA). Total RNA was isolated using TRIzol™ reagent per the manufacturer’s instructions. Approximately 1 μg of each sample was primed with oligo (dT)18 and random hexamer primers prior using Maxima First Strand cDNA synthesis kit. For quantitative real-time RT-PCR, targets were amplified with Maxima SYBR Green/ROX qPCR Master Mix on an Applied Biosystems ViiA 7 Real-Time PCR System (Foster City, CA, USA) using gene-specific primer pairs (IL1β: forward 5′-GGA GAA TGA CCT GAG CAC CT-3′; reverse 5′-GGA GGT GGA GAG CTT TCA GT-3′, IL-6: forward 5′-AGT CCT GAT CCA GTT CCT GC-3′; reverse 5′-AAG CTG CGC AGA ATG AGA TG-3′, with normalization to the housekeeping GAPDH: forward 5′-CGA CCA CTT TGT CAA GCT CA-3′; reverse 5′-AGG GGT CTA CAT GGC AAC TG-3′). Relative gene expression was determined using the 2^-ΔΔCT^ method [38].

### Statistical analyses

Statistical analyses were performed using the SPSS statistics software (version 21, IBM, Armonk, NY, USA). Kruskal-Wallis analyses of variance (ANOVA) with Dunn’s post hoc tests and chi-square tests were used to compare the characteristics of the cases and control groups. Significantly altered plasma S1P species would then be stratified into tertiles and subject to multivariate analyses using binary logistic regression, with odds ratios (OR) and 95% confidence intervals (95% CI) computed for CIND, AD, and VaD as outcomes. Models were adjusted for age, education, APOE ε4 carrier status, hypertension, diabetes, and heart disease as covariates, as these variables were significantly different between groups (see Table 2). To examine the associations between S1P and pro-inflammatory cytokines, general linear models were performed with each log-transformed S1P level included as a determinant while each log-transformed cytokine level was defined as outcomes. Similarly, the models were adjusted for age, hypertension, hyperlipidemia, diabetes, and heart disease as covariates. For cell culture experiments, S1P-treated groups were compared with control and with one another using one-way ANOVA with Bonferroni’s correction for multiple comparisons. For all analyses, *P* values < 0.05 were considered statistically significant.

**Table 2:** Baseline characteristics and plasma S1P levels of participants based on to their cognitive groups (*n* = 384)

Characteristics	NCI (***n*** = 80)	CIND (***n*** = 160)	AD (***n*** = 113)	VaD (***n*** = 31)	***p*** value
Demographic and disease variables					
Age, years, mean (SD)	68.8 (6.3)	72.0 (8.1)*	76.2 (7.7)*†	72.9 (8.8)	**< 0.001**
Female, no.(%)	41 (51.3)	80 (50.0)	77 (68.1)	11 (35.5)	**0.002**
Education ≤ primary, no.(%)	25 (31.3)	83 (51.9)	30 (26.6)	14 (45.2)	**< 0.001**
APOE4 carrier, no.(%)	14 (17.5)	49 (30.6)	40 (35.4)	8 (25.8)	0.051
Hypertension, no.(%)	45 (56.3)	109 (68.1)	90 (79.7)	31 (100.0)	**< 0.001**
Diabetes, no.(%)	18 (22.5)	55 (34.4)	47 (41.6)	16 (51.6)	**0.01**
Cardiovascular disease, no.(%)	5 (6.3)	26 (16.3)	21 (18.6)	12 (38.7)	**0.001**
Hyperlipidemia, no.(%)	54 (67.5)	121 (75.6)	82 (72.6)	29 (93.6)	**0.04**
Blood-based markers					
IL-6, pg/mL, median (IQR)	0.79 (2.5)	1.10 (2.4)	1.59 (2.3)	1.75 (2.9)	0.063
IL-8, pg/mL, median (IQR)	4.48 (1.9)	5.20 (3.6)^‡^	5.65 (4.0)^‡^	5.68 (4.7)^‡^	**< 0.001**
TNF, pg/mL, median (IQR)	4.05 (2.9)	4.47 (2.8)	5.28 (3.5)^‡§^	5.98 (4.0)^‡§^	**< 0.001**
d16:1 S1P, nM, median (IQR)	58.2 (31)	55.4 (25)^‡^	53.1 (24)^‡^	49.9 (14)^‡§^	**0.007**
d17:1 S1P, nM, median (IQR)	17.4 (5.4)	16.6 (6.0)	15.7 (6.5)	16.2 (4.2)	0.486
d18:0 S1P, nM, median (IQR)	60.1 (19.9)	60.9 (20.1)	62.5 (21.0)	66.1 (29.5)	0.607
d18:1 S1P, nM, median (IQR)	483.2 (134)	484.9 (157)	483.1 (189)	582.2 (256)	0.127
d18:1 to d16:1 ratio, median (IQR)	8.5 (3.9)	9.6 (5.0)^‡^	9.8 (5.3)^‡^	11.5 (5.2)^‡§#^	**< 0.001**

Bold texts indicate *p* values for significant tests by one-way ANOVA (age); chi-square (gender, primary education, APOE carrier, hypertension, diabetes, cardiovascular disease, hyperlipidemia); Kruskal-Wallis ANOVA (blood-based markers)

*NCI* non-cognitive impairment, *CIND* cognitive impairment no dementia, *S1P* sphingosine-1-phosphate, *n* number of cases, *SD* standard deviation, *IQR* interquartile range

*Significantly different from NCI (one-way ANOVA with post hoc Bonferroni tests *p* < 0.05)

^†^Significantly different from CIND (one-way ANOVA with post hoc Bonferroni tests *p* < 0.05)

^#^Significant chi-square tests (*p* < 0.05)

^‡^Significantly different from NCI (Kruskal-Wallis ANOVA with post hoc Dunn’s tests *p* < 0.05)

^§^Significantly different from CIND (Kruskal-Wallis ANOVA with post hoc Dunn’s tests *p* < 0.05)

^#^Significantly different from AD (Kruskal-Wallis ANOVA with post hoc Dunn’s tests *p* < 0.05)

## Results

### Demographic characteristics and disease factors in cognitive subgroups

A total of 459 subjects were recruited from August 2010 to July 2015, of which 384 had sufficient baseline plasma available for biomarker analysis (see Supplementary Figure 1). In the current study, baseline diagnoses of subjects were 80 (20.8%) non-cognitively impaired (NCI), while the cognitive subgroups were 150 (39.1%) CIND, 113 (29.4%) AD, and 31 (8.1%) VaD. Table 2 shows the baseline demographic characteristics and plasma S1P levels of the study cohort. In line with previous studies of this cohort [29, 39], cases were significantly older, had lower education, and had higher prevalence of ≥ 1 APOE4, as well as more hypertension, diabetes, and cardiovascular disease as compared to NCI.

### Altered peripheral inflammatory and S1P markers in cognitive subgroups

**Fig. 1:**
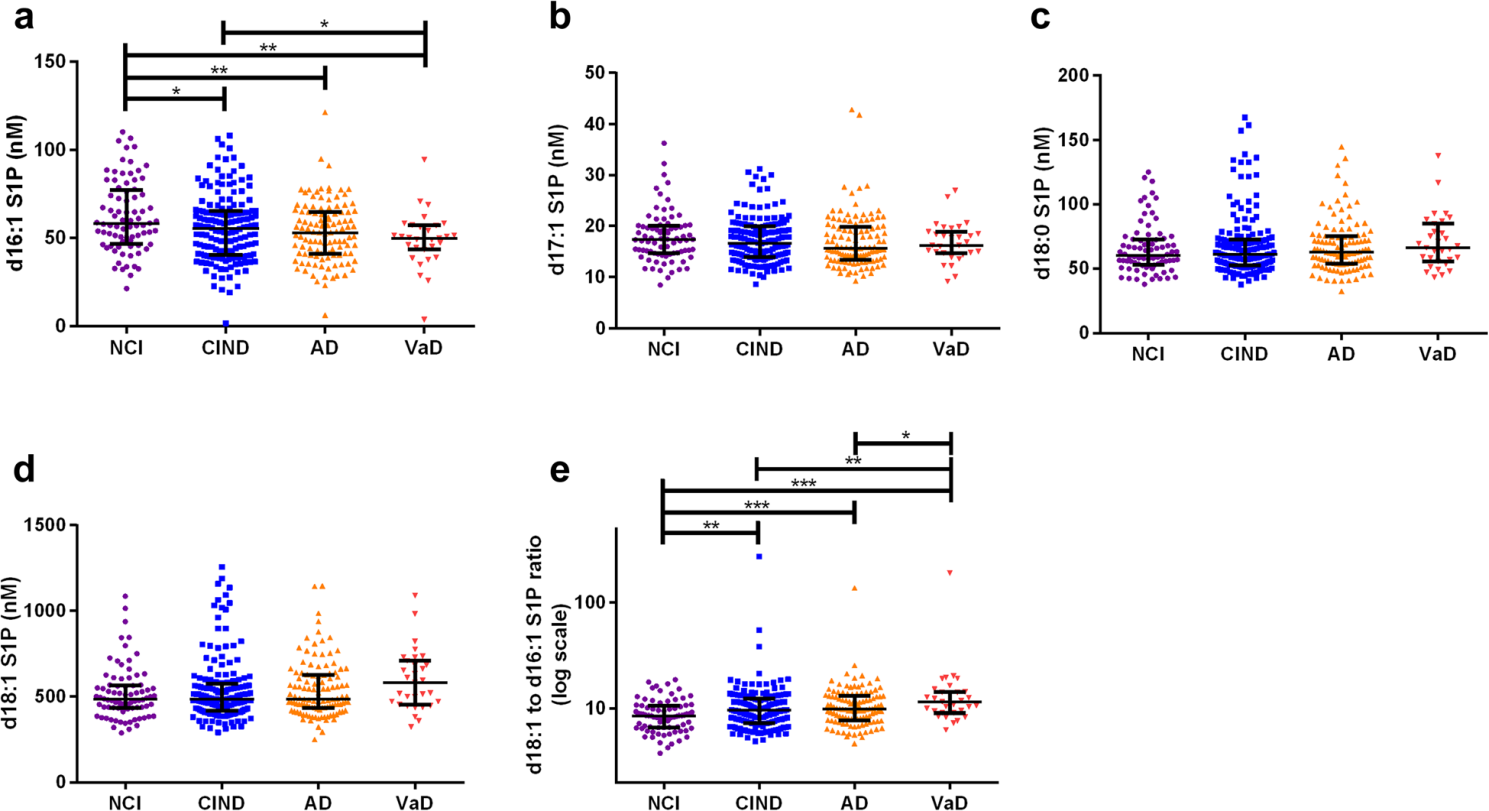
Plasma d16:1 S1P and S1P ratios were altered in a cohort of dementia patients. **a** d16:1 S1P, **b** d17:1 S1P, **c** d18:0 S1P, and **d** d18:1 S1P in nM were plotted across the diagnosis groups. **e** d18:1 to d16:1 S1P ratio was plotted across diagnosis groups and shown on a log-10 scale for better visualization of the spread of datapoints. Bars are expressed as median ± IQR. Pairwise comparisons were determined by Kruskal-Wallis test followed by post hoc Dunn’s tests, *p < 0.05, **p < 0.01, ***p < 0.001

With respect to peripheral cytokines and S1P levels, Table 2 shows that IL-8 levels were significantly increased in all cognitive subgroups, while TNF levels were raised only in AD and VaD. In contrast, d16:1 S1P levels were significantly lower in all cognitive subgroups, while ratios of d18:1 to d16:1 S1P were significantly higher in all cognitive subgroups, with the highest values found in the VaD group (significantly higher than CIND and AD by Kruskal-Wallis ANOVA with Dunn’s post hoc test, *p* < 0.05, see Table 2, Fig. 1a, e).

**Fig. 2:**
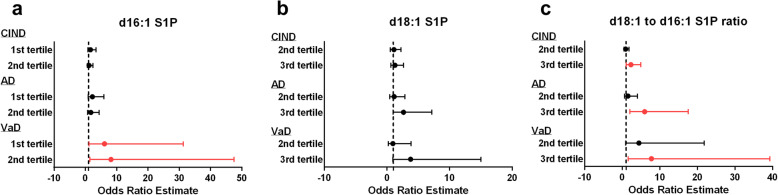
Forest plot summary of logistic regression analyses for CIND, AD, and VaD risk. **a** 1st and 2nd tertiles of d16:1 S1P were significantly associated with higher risk of VaD, **b** d18:1 S1P tertiles were not significantly associated with risk of disease, and **c** 3rd tertile of d18:1 to d16:1 S1P ratio was significantly associated with higher risk of CIND, AD, and VaD. Dotted line represents odds ratio of 1. Dot represents odds ratio (OR) and the line represents corresponding 95% confidence intervals (95% CI). Dots and lines in red represent significant association

Table 3 shows the multivariate analyses of associations between significantly altered S1P markers and cognitive subgroups (described as odds ratios [OR] of being in each cognitive subgroup for tertile-segregated S1P values, with OR values for the VaD subgroup estimated using the Firth method [40, 41] due to the presence of hypertension covariate in all VaD subjects). These results are also summarized in Fig. 2, a forest plot showing the risk of CIND, AD, and VaD for each tertile of S1P species. Compared to the highest tertile of d16:1, the lowest and middle tertiles were associated with VaD, but not CIND or AD (Fig. 2a). In contrast, while d18:1 itself showed no significant associations, the highest tertile for d18:1 to C16:1 ratio was associated with the CIND, AD, and VaD subgroups (Fig. 2c). These associations remain significant even after adjusting for unmatched covariates of age, gender, education, presence of APOE4 allele, hypertension, diabetes, hyperlipidemia, and cardiovascular disease (from Table 2).

**Table 3:** Association between S1P (tertiles) and cognitive impairment, expressed as odds ratios (OR) and 95% confidence intervals (CI)

	CIND (***n*** = 160), OR (95% CI)*	AD (***n*** = 113), OR (95% CI)*	VaD (***n*** = 31), OR (95% CI)*^#^
d16:1 S1P			
1st tertile	1.63 (0.79–3.38)	2.24 (0.85–5.89)	**6.12 (1.20–31.33)**
2nd tertile	1.18 (0.59–2.37)	1.74 (0.69–4.39)	**8.19 (1.41–47.56)**
3rd tertile	1	1	1
d18:1 S1P			
1st tertile	1	1	1
2nd tertile	1.10 (0.54–2.22)	1.14 (0.45–2.88)	0.94 (0.23–3.84)
3rd tertile	1.28 (0.63–2.63)	2.64 (0.97–7.16)	3.78 (0.95–14.99)
d18:1 to d16:1 ratio			
1st tertile	1	1	1
2nd tertile	0.91 (0.45–1.83)	1.51 (0.57–4.01)	4.43 (0.90–21.80)
3rd tertile	**2.30 (1.07–4.94)**	**5.97 (2.03–17.54)**	**7.75 (1.53–39.28)**

*CIND* cognitive impairment no dementia, *AD* Alzheimer’s disease, *VaD* vascular dementia, *S1P* sphingosine-1-phosphate, *OR* odds ratio, *CI* confidence interval

*Adjusted for age, gender, education, presence of APOE4 allele, hypertension, diabetes, hyperlipidemia, and cardiovascular disease

^#^Estimated using Firth’s method due to separation where hypertension perfectly predicts VaD

### d18:1 S1P and d18:1 to d16:1 ratio are associated with peripheral inflammatory markers

Based on our a priori hypothesis that S1Ps function as immunomodulators, further regression analyses using general linear models were performed between significantly altered S1P markers and pro-inflammatory cytokines (log-transformed values of IL-6, IL-8, and TNF concentrations). Out of the 382 subjects with available measurements, IL-6 was below the detection limit for 106 subjects, namely 27 (33.8%) NCI, 50 (31.3%) CIND, 21 (18.6%) AD, and 8 (25.8%) VaD, while TNF was below the detection limit for 3 subjects, namely 2 (2.5%) NCI and 1 (0.6%) CIND. In these cases, the lowest detectable values specified by the manufacturer were used in statistical analyses (0.2 pg/mL for IL-6; 0.3 pg/mL for TNF). Table 4 shows that after adjusting for risk factors, every 1% increase in d18:1 S1P levels was significantly associated with a 0.79% increase in IL-6 (95% CI = 0.32 to 1.26) and 0.28% increase in TNF (95% CI = 0.07 to 0.49). Similarly, increased d18:1 to d16:1 S1P ratio was significantly associated with increases in all three inflammatory markers (β for IL-6 = 0.38, 95% CI = 0.08 to 0.68; β for IL-8 = 0.14, 95% CI = 0.03 to 0.25; β for TNF = 0.16, 95% CI = 0.03 to 0.29). In contrast, no significant associations were found between d16:1 S1P levels and the inflammatory markers measured. In order to avoid potential analytic bias and floor effect due to the substitution of values outside the detection range with the minimal detection points as stated above, linear regression analyses were repeated after removing data points outside of detection ranges. After excluding the said data points (*n* = 106 for IL-6 and *n* = 3 for TNF), the associations of d18:1 to d16:1 S1P ratio with IL-6 and TNF remained significant (β for IL-6 = 0.33, 95% CI = 0.05 to 0.61; β for TNF = 0.15, 95% CI = 0.03 to 0.27).

**Table 4:** Linear regression of log-transformed plasma S1P and serum inflammatory markers

	IL-6*, β (95% CI)^**†**^	IL-8*, β (95% CI)^**†**^	TNF*, β (95% CI)^**†**^
d16:1 S1P*	− 0.07 (−0.40, 0.26)	− 0.12 (−0.24, 0.01)	− 0.06 (− 0.20, 0.09)
d18:1 S1P*	**0.79 (0.32, 1.26)**	0.10 (−0.07, 0.28)	**0.28 (0.07, 0.49)**
d18:1 to d16:1 ratio*	**0.38 (0.08, 0.68)**	**0.14 (0.03, 0.25)**	**0.16 (0.03, 0.29)**

*IL-6* interleukin-6, *IL-8* interleukin-8, *TNF* tumor necrotic factor, *S1P* sphingosine-1-phosphate, *β* mean difference, *CI* confidence interval

*Log-transformed

^†^Adjusted for age, hypertension, hyperlipidemia, diabetes, and cardiovascular disease. Interpretation: A 1% increase in C18:1 to C16:1 ratio will give rise to β% increase in cytokines (IL-6, IL-8, or TNF) serum levels

### d16:1 S1P attenuates d18:1 S1P-induced cytokine gene expression

**Fig. 3:**
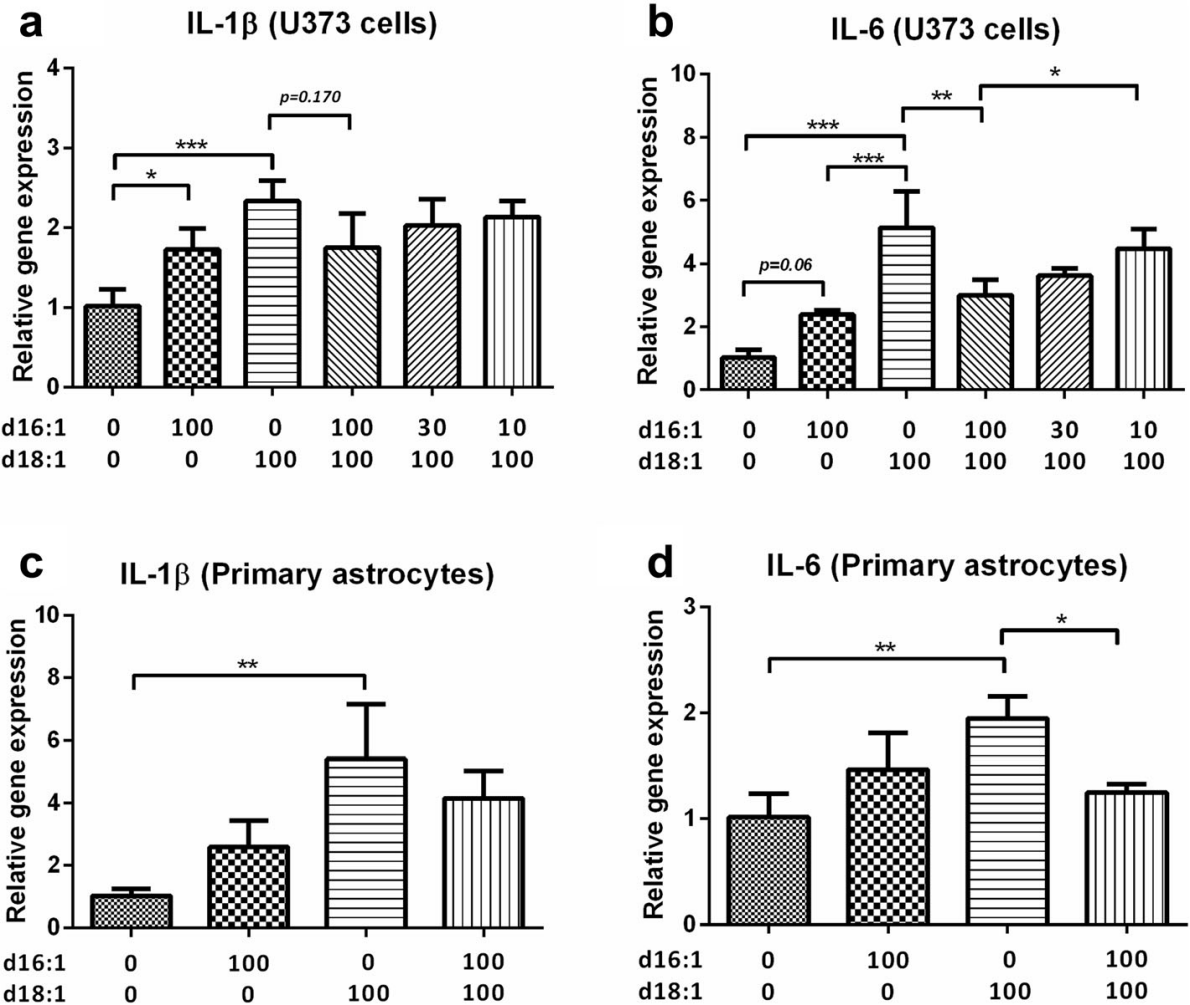
Attenuation of d18:1 S1P-induced cytokine gene expression by d16:1 S1P. U-373 MG cells (**a**, **b**) and mouse primary astrocytes (**c**, **d**) were co-treated with d18:1 S1P and d16:1 S1P at the concentrations indicated on the graphs (in nM) and then evaluated for gene expression of IL-1β (**a**, **c**) and IL-6 (**b**, **d**) by real-time qPCR. Pairwise comparisons were determined by one-way ANOVA followed by Bonferroni’s post hoc tests, *p < 0.05, **p < 0.01, ***p < 0.001 with *N* = 3–4 independent experiments

Since it has been shown that S1Ps can upregulate expression of pro-inflammatory cytokines in astrocytes [42, 43], we studied the effects of d16:1 vs. d18:1 S1P in vitro using both mouse primary astrocytes as well as an astroglial cell line (U-373MG). Figure 3 shows that treatment with either d16:1 or d18:1 S1P resulted in significant increases in mRNA for two acute-phase inflammatory cytokines, IL-1β and IL-6, relative to vehicle-treated control cells. Interestingly, the extent of gene induction by d16:1 S1P was lower than those by d18:1 S1P at equal concentrations in both primary astrocytes and U-373MG cells. Furthermore, when U-373MG cells were co-treated with d16:1 and d18:1 S1P, the induction of cytokine genes by d18:1 S1P was attenuated by d16:1 S1P in a dose-dependent manner (Fig. 3a, b). This trend was seen with both IL1ß and IL-6, but reached statistical significance only with the latter (Fig. 3b), likely due to the larger overall magnitude of the effect. To ensure that the effect was not specific to this cell line, the experiment was repeated using primary mouse cortical astrocytes (Fig. 3c, d), where we again observed the attenuation of d18:1 S1P-mediated cytokine gene induction when co-incubated with d16:1 S1P, reaching statistical significance for IL-6 (Fig. 3d).

## Discussion

Bioactive sphingolipids are now known to mediate signaling in diverse cellular processes and are gaining recognition for their roles in health and disease as well as potential utility as therapeutic targets and clinical biomarkers for various conditions including cancer, neurodegenerative, metabolic, autoimmune. and vascular diseases [25, 44–50]. In the current study, we observed alterations of plasma sphingosine-1-phosphate (S1P) species in a cohort of aged cognitively normal subjects as well as people with vascular cognitive impairment (VCI) and Alzheimer’s disease (AD). Firstly, we found specific reductions of d16:1 S1P in VaD. Since this change was observed only in the quantitatively minor d16:1 species and not in global S1P content, it is likely to be due to pathophysiology specific to VaD rather than a result of general dyslipidemia. In addition, our MS study utilized a new derivatization method demonstrated to enhance sensitivity in the quantification of S1P [24]. This improved sensitivity likely conferred the capability to detect changes in the less abundant, non-canonical S1P species, one of which we have now shown to be altered in VaD.

A second major finding was higher d18:1 to d16:1 ratios in all cognitive subgroups (CIND, VaD, AD) studied. Furthermore, d18:1 to d16:1 ratios were positively correlated with cytokines IL-6, IL-8, and TNF, in line with previous studies by ourselves and others demonstrating immunomodulatory functions of S1P [42, 43, 51]. Taken together, our findings not only support further assessments of the clinical utility of d16:1 S1P levels as a potential diagnostic biomarker for VaD, but also help advance the notion of perturbations in S1P-regulated inflammatory responses as novel mechanisms underlying both AD and VCI. Furthermore, because chronic neuroinflammation is a well-established pathogenic factor for both AD and CeVD [9–11], our data also support further assessment of S1P (as well as their receptors and associated metabolic pathways) as potential therapeutic targets for these conditions.

While S1P and related sphingolipids have previously been implicated in AD and CeVD [11, 44, 47, 48, 52, 53], detailed mechanisms underlying their involvement remain unclear. Cumulative evidence suggests that S1P likely plays a net pro-inflammatory role in the CNS, primarily mediated by the astrocytes and vascular endothelial cells. Previous reports have demonstrated that S1P signaling can cause astrogliosis and upregulation of inflammatory mediators, such as IL1β, IL-6, and VEGF [42, 54]. It is possible that dysregulation of S1P signaling can lead to prolonged, chronic elevation of inflammation. Interestingly, S1P is present in human plasma at levels that are well above the EC_50_ of its cognate receptors. Since ligand availability is not a limiting factor, it is unclear how S1P receptor activity is regulated. This regulation is likely to involve ligand sequestration/differential presentation by carrier molecules [55, 56] and rapid metabolism in tissue parenchyma that results in regulation of local concentrations [57].

In order to further elucidate the mechanisms underlying associations of S1P with inflammation, we followed up on the clinical observations with in vitro studies using S1P-induced gene expression of acute-phase cytokines in primary astrocytes and astroglial cell line U373-MG. Interestingly, for both S1P-inducible [54] IL-1β and IL-6, the effects on cytokine gene upregulation were higher for d18:1 than for d16:1 S1P. The differential potency of d16:1 vs. d18:1 S1P has previously been shown in cell-based assays [58, 59]. Here, the relatively weak effects of d16:1 S1P on cytokine gene induction may account for the lack of correlations between d16:1 and cytokine levels in the clinical cohort (see Table 4). Furthermore, co-treatment with equimolar concentrations of d18:1 and d16:1 S1P attenuated the extent of d18:1-induced cytokine gene upregulation, with subsequent reductions of d16:1 S1P concentration (to simulate clinical observations) also reducing the attenuation (i.e., de-repression of cytokine gene induction, see Fig. 3c, d). Taken together, our data indicate that d16:1 S1P is a partial agonist for cytokine gene induction responses but acts as a functional antagonist by attenuating the effects of full agonist d18:1 when both S1P species are present in the same milieu. These in vitro findings led us to postulate that d16:1 S1P may function physiologically to “fine-tune” the pro-inflammatory effects of d18:1. We therefore proposed further clinical evaluation of the d18:1 to d16:1 S1P ratio as a parameter to that evaluates the net effect of S1P-mediated neuroinflammation, thus accounting for the potential interaction between the two S1P species. The potential clinical utility of the proposed parameter is supported by the positive association between this parameter and both cognitive subgroups as well as peripheral cytokine levels (see above). This further suggests that any dysregulation of S1P-mediated immunomodulation which tips towards a pro-inflammatory course (in this case due primarily to decreased levels of the functional antagonist d16:1 leading to de-repression of cytokine induction) may contribute to AD and VCI pathophysiology. However, our findings and interpretations are subject to several limitations described below.

### Limitations

First, the postulate that dysregulated S1P-mediated immunomodulation is involved in AD and VCI is necessarily an over-simplification, as the myriad S1P species as well as receptors and signaling partners indicate a much more complicated picture for S1P-mediated immunomodulation. A case in point is the recent study by Vutukuri et al. [60] showing functional antagonism by d20:1 S1P on d18:1-mediated induction of cyclooxygenase (COX)-2 expression. As another example, we have not studied in detail the other two S1P species we measured (d17:1, d18:0) when they did not show significant alterations at the univariate analyses stage (see Table 2, Fig. 1b, c), but their potential interactions with d16:1 and d18:1 cannot be ruled out, and further studies are required to carefully delineate the potential roles of d17:1, d18:0, d20:1, and multiple other S1P species in AD and VCI.

In addition to the limitation described above, other molecules and pathways mediating S1P signaling have not been well studied. One important research gap is the elucidation of the specific S1P receptor subtypes mediating the immunomodulatory effects of d16:1 and d18:1 S1P. Previous work by others has suggested the potential involvement of S1PR_2_ and S1PR_3_ [42, 60], while we have shown recently that d16:1 S1P has a lower potency for S1PR_2_ and a lower efficacy for S1PR_3_ [58]. Increasing d16:1 S1P concentrations would result in a reduction of pro-inflammatory S1PR_3_ signaling, while having an additive effect on S1PR_1_ and a minimal effect on S1PR_2_, thus indicating that the partial agonist effects of d16:1 S1P are receptor isoform-dependent. This adds another layer of complexity to potential pathways and ligand interactions underlying S1P-mediated immunomodulation which require further study, especially in the context of neurodegeneration and CeVD. Furthermore, the clinical correlates of the observed S1P alterations, especially the measures relevant to AD and VCI (e.g., brain atrophy and CeVD), are at present unknown. Also, we are currently unable to conclude whether the observed S1P alterations reflect pathogenic brain perturbations or are a sequela of VCI. Interestingly, S1P signaling to endothelial cells and astrocytes are known to regulate blood-brain barrier (BBB) integrity [61], leading to speculation that S1P perturbations which result in BBB defects may also underlie their utility as biomarkers, due to CNS S1P leakage into the systemic circulation. However, this postulate remains to be verified. In vivo studies have shown that S1P is altered in the CNS in VCI; for example, animal models for VCI, including the BCAS and MCAO mice models, have altered brain S1P levels as well as S1P-associated enzymes [62, 63]. Hence, it is likely that our observations could reflect what is happening within the CNS. It is also possible that the alterations in peripheral S1P may also be contributed by circulating erythrocytes and endothelial cells, which are established sources of peripheral S1P [64]. One of the important functions of S1P is in modulating vascular biology; for example, it has been demonstrated that S1P is involved in endothelial barrier integrity, and vascular smooth muscle function [51, 65]. Peripheral and CSF S1P has also been found to be altered in stenosis and stroke, a key event prior to onset of VCI [66] as well as in AD [67]. It is likely that peripheral S1P may also be affecting the CNS in indirect ways. Taken together, the plasma S1P levels we quantified may be possibly contributed and influenced by both peripheral and CNS changes, and we are currently unable to tease out the S1P derived only from the CNS itself.

Lastly, from a clinical research perspective, our findings from a case-control study design represent initial steps in advancing S1P as potentially novel biomarkers for AD and VCI but require larger cohort and longitudinal approaches to validate their clinical utility. Furthermore, while we investigated the involvement of S1P and its immunoregulatory roles in the two commonest forms of dementia (AD and VaD), it is probable that S1P may be of relevance to other neurodegenerative disorders whereby neuroinflammation is a suspected factor and player, for example, in Lewy Body Dementias and Frontotemporal Dementia. Future studies may explore the involvement of S1P in these diseases.

## Conclusions

To our knowledge, this report is the first to identify alterations in peripheral non-canonical S1P species (d16:1) which are associated with a VCI subgroup, namely VaD. The findings support the use of d16:1 S1P as a potential diagnostic biomarker for VaD and the d18:1 to d16:1 ratio as an index of dysregulation of S1P-mediated immunomodulation leading to an inflammatory state. Therefore, our study also identifies S1P-mediated pathways as novel therapeutic targets to ameliorate chronic neuroinflammation which may contribute to, or exacerbate, AD and VCI. However, further clinical and basic research is needed to validate clinical utility as biomarkers and to elucidate the complex pathobiology of S1P and related sphingolipids in AD and VCI.

## Supplementary information

**Additional file 1: Supplementary Figure 1.** Schematic of subject recruitment for study.

**Additional file 2: Supplementary Information.** Synthesis of S1P d16:1.

## Data Availability

The data that support the findings of this study are available from the corresponding authors, DRH or MKPL, upon reasonable request.

## Abbreviations

AD: Alzheimer’s disease
ANOVA: Analysis of variance
APOE: Apolipoprotein E
BBB: Blood-brain barrier
CeVD: Cerebrovascular disease
CI: Confidence intervals
CIND: Cognitive impairment, no dementia
DMEM: Dulbecco’s modified Eagle’s medium
EC50: Half maximal effective concentration (measure of potency)
EDTA: Ethylenediaminetetraacetic acid
FBS: Fetal bovine serum
GAPDH: Glyceraldehyde 3-phosphate dehydrogenase
GFAP: Glial fibrillary acidic protein
HBSS: Hank’s Balanced Salt Solution
HILIC: Hydrophilic interactions chromatography
HPLC: High-performance liquid chromatography
IL-1β: Interleukin-1-beta
IL-6: Interleukin-6
IL-8: Interleukin-8
IQR: Interquartile range
ISTD: Internal standards
LC-MS/MS: Liquid chromatographic-tandem mass spectrometry
mRNA: Messenger RNA
NCI: Non-cognitively impaired
NINCDS-ADRDA: National Institute of Neurological and Communicative Disorders and Stroke and the Alzheimer’s disease and Related Disorders Association
NINDS-AIREN: The National Institute of Neurological Disorders and Stroke-Association Internationale pour la Recherché et l’ Enseignement en Neuroscience
OR: Odds ratio
PCA: Principal components analysis
RT-PCR: Quantitative reverse transcriptase polymerase chain reaction
S1P: Sphingosine-1-phosphate
S1PR: Sphingosine-1-phosphate receptor
SD: Standard deviation
SST: Serum-separating tubes
TNF: Tumor necrosis factor
VaD: Vascular dementia
VCI: Vascular cognitive impairment
VEGF: Vascular endothelial growth factor
