# Supplementary figures and images for: Immunomodulatory sphingosine-1-phosphates as plasma biomarkers of Alzheimer’s disease and vascular cognitive impairment
Source: Alzheimers Res Ther. 2020 Sep 30;12:122. doi: 10.1186/s13195-020-00694-3 (PMC7528375; doi:10.1186/s13195-020-00694-3)

**Chua et al Supplementary Figure 1: Schematic of subject recruitment for study**

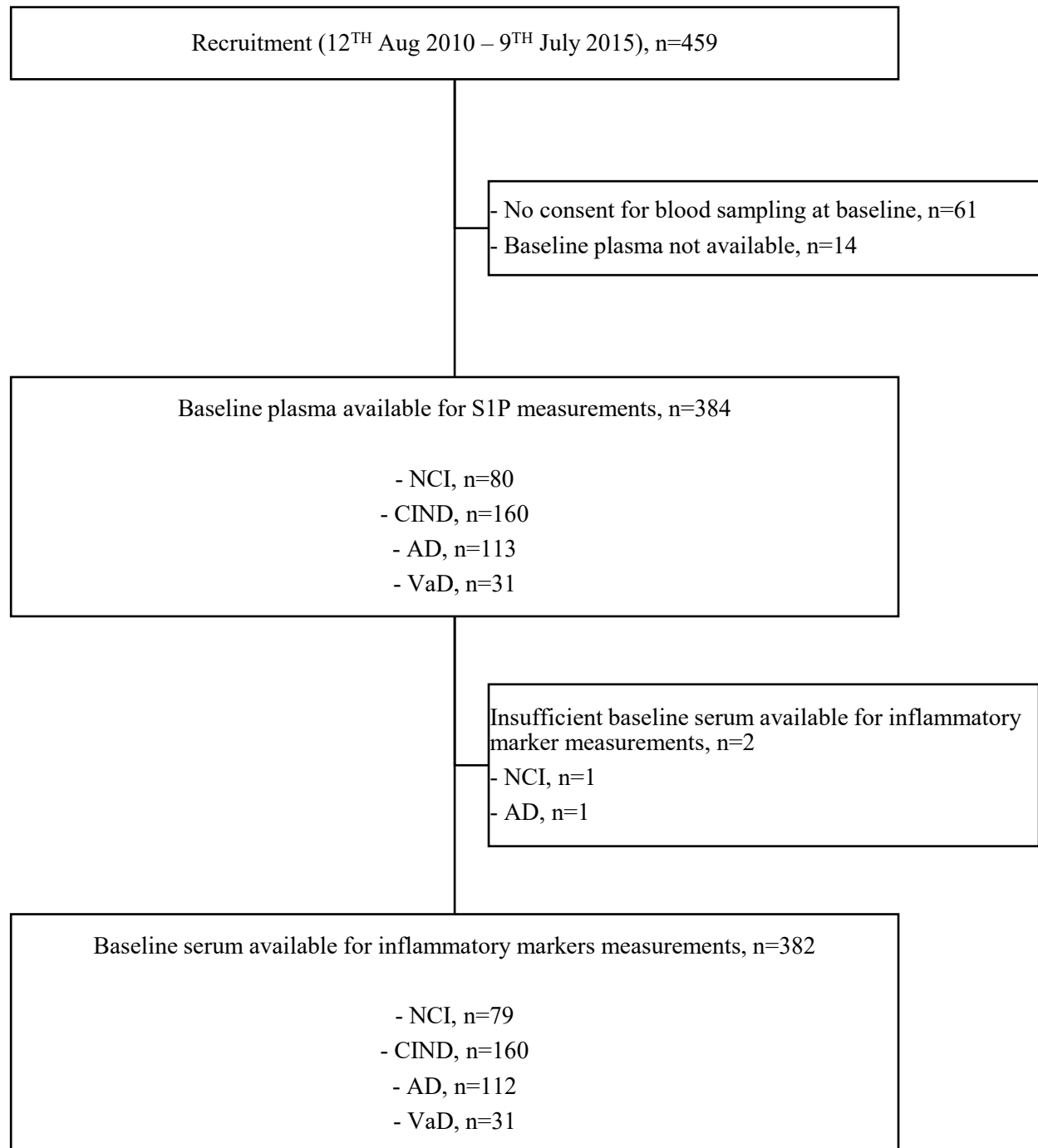

Supplement: Supplementary file 1 — Additional file 1: Supplementary Figure 1. Schematic of subject recruitment for study. [file 13195_2020_694_MOESM1_ESM.pdf]
