# Supplementary material for: Immunomodulatory sphingosine-1-phosphates as plasma biomarkers of Alzheimer’s disease and vascular cognitive impairment
Source: Alzheimers Res Ther. 2020 Sep 30;12:122. doi: 10.1186/s13195-020-00694-3 (PMC7528375; doi:10.1186/s13195-020-00694-3)
Supplement: Supplementary file 2 — Additional file 2: Supplementary Information. Synthesis of S1P d16:1. [file 13195_2020_694_MOESM2_ESM.pdf]

# Synthesis of S1P d16:1

**Amy R. Howell & Kaddy Camara**
**Department of Chemistry, University of Connecticut, Storrs, CT, 06269-3060 USA**

**General Experimental.** Tetrahydrofuran (THF) was dried using a solvent dispensing system (SDS) with a column of neutral alumina. Pyridine, toluene, dimethylformamide (DMF), methylene chloride ($CH_2Cl_2$), deuterated chloroform ($CDCl_3$), methanol (MeOH), deuterated methanol ($CD_3OD$) and ethanol (EtOH) were dried over 4Å molecular sieves (MS). *N*-*tert*-Boc-*L*-serine was purchased from Novabiochem and TCI, respectively. The other reagents were purchased from Acros, Alfa Aesar or Aldrich and used without further purification. (2*S*)-*N*-methoxy-*N*-methyl 2-(*N*-*tert*-butyloxycarbonylamino)-propanamide (**1**) was prepared as described in the literature.[1] All reactions were conducted under an atmosphere of $N_2$ in glassware that had been dried overnight in an oven at 120 °C. Where appropriate, control of the reaction temperature was achieved with a solid $CO_2$/acetone bath, an ice bath or a heated oil bath.

$^1$H NMR spectra were recorded at 400 MHz and calibrated to the residual $CHCl_3$ peak in $CDCl_3$ at 7.26 ppm, to the TMS peak at 0.0 ppm or to the residual $CD_3OH$ peak in $CD_3OD$ at 3.34 ppm. $^{13}$C NMR spectra were recorded at 100 MHz and calibrated to the residual $CHCl_3$ peak in $CDCl_3$ at 77.23 or to the residual MeOH peak in MeOD at 49.5 ppm. $^{31}$P NMR spectra were recorded at 162 MHz. The following abbreviations are used for peak multiplicities: app (apparent), s (singlet); br s (broadened singlet); d (doublet); dd (doublet of doublet); ddd (doublet of doublet of doublets); dddd (doublet of doublet of doublet of doublets); dt (doublet of triplets); tt (triplet of triplets) t (triplet); q (quartet); quin (quintet); m (multiplet). Coupling constants, *J*, are reported in Hertz (Hz).

IR spectra were recorded on a Brucker FT-IR spectrometer. High-resolution mass spectra (HRMS) were obtained on an AccuTOF instrument equipped with a DART ionization source. Melting points were observed in open Pyrex capillary tubes and are uncorrected. Specific rotations $[\alpha]_D$ were obtained on a JASCO polarimeter using the sodium D-line as a source, and the concentration (c) is expressed in g per 100 mL. Flash column chromatography was performed on Silica Gel, 40 micron, 32-63 flash silica from Sorbent. Thin layer chromatography (TLC) was performed on silica gel (Silicycle Silica Gel 60 $F_{254}$ glass plates). Compounds were visualized on the TLC plates by UV, 5% phosphomolybdic acid in ethanol, 0.5% potassium permanganate in water or a solution of ethanol/$H_2SO_4$/AcOH/*p*-anisaldehyde (135:5:1.5:3.7).

## Scheme: Preparation of S1P d16:1

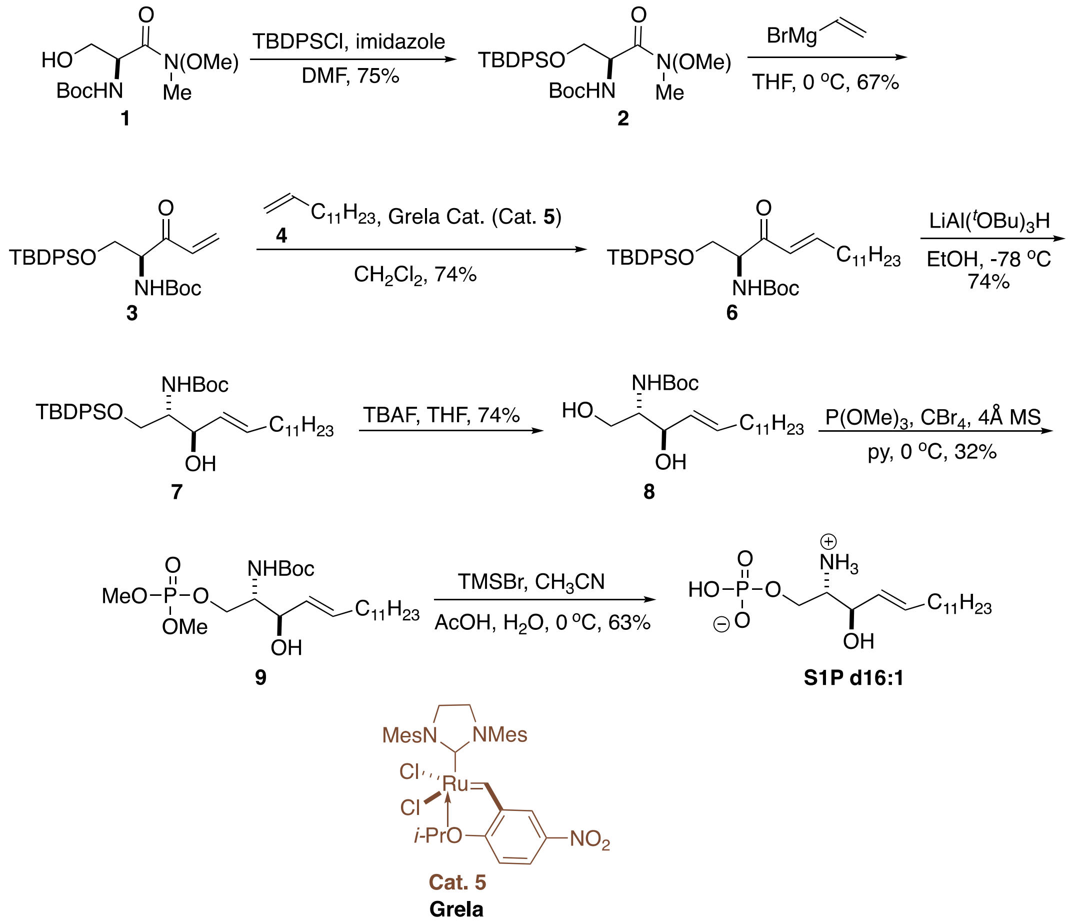


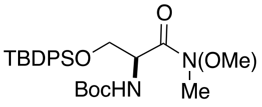

**2**

***N*-Methoxy-*N*-methyl (2*S*)-3-(*tert*-butyldiphenylsilyloxy)-2-(*N*-*tert*-butyloxycarbonyl amino)propanamide (2).** Imidazole (3.18 g, 46.7 mmol) was added to solution of (2*S*)-*N*-methoxy-*N*-methyl 2-(*N*-*tert*-butyloxycarbonylamino)propanamide[1] (**1**) (3.86 g, 15.6 mmol) in dry DMF (10 mL). TBDPSCl (6.0 mL, 23 mmol) was then added, and the reaction was stirred overnight. $H_2O$ (200 mL) was added, and the solution was extracted with $Et_2O$ (3 X 100 mL). The combined organic extracts were dried ($MgSO_4$), filtered and concentrated. The crude product was purified using flash column chromatography on silica gel (petroleum ether/EtOAc, 95:5) to give **2** (5.68 g, 75%) as a colorless oil: $[\alpha]^{25}_{D}$ 11.00 (*c* 1.09, $CH_2Cl_2$); IR (neat) 2932, 1713, 1664, 1427, 1365, 1249, 1166, 1108, 733, 701, 503 $cm^{-1}$; $^1H$ NMR (400 MHz, $CDCl_3$) δ 7.64 (tt, *J* = 5.9, 1.5 Hz, 4H), 7.44–7.35 (m, 6H), 5.41 (d, *J* = 8.7 Hz, 1H), 4.84 (app dd, *J* = 4.2, 4.2 Hz, 1H), 3.87

(app d, $J$ = 4.8 Hz, 2H), 3.66 (m 3H), 3.19 (s, 3H), 1.44 (s, 9H), 1.04 (s, 9H); $^{13}$C NMR (100 MHz, $CDCl_3$) δ 170.9, 155.6, 135.8, 133.4, 129.9, 127.9, 79.7, 64.3, 61.6, 52.7, 32.4, 28.6, 26.9, 19.4; HRMS (ESI) calcd for $C_{26}H_{39}N_2O_5Si$ [M + H]$^+$ *m/z* 487.2623, found 487.2632.

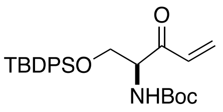

**3**

**(2*S*)-1-(*tert*-Butyldiphenylsilyloxy)-2-(*N*-*tert*-butyloxycarbonylamino)-4-penten-3-one (3).** Vinyl magnesium bromide (1 M in THF, 95 mL, 95 mmol) was added drop-wise via an addition funnel to a solution of (2*S*)-*N*-methoxy-*N*-methyl 3-(*tert*-butyldiphenylsilyloxy)-2-(*N*-*tert*-butyloxycarbonylamino)propanamide (**2**) (11.5 g, 23.7 mmol) in THF (70 mL) at 0 °C. The reaction was allowed to slowly warm to rt and to stir for 1.5 h. The solution was cooled to 0 °C, and then HCl (aqueous 10%, 50 mL) was added. The solution was extracted with EtOAc (3 X 50 mL). The combined organic extracts were dried ($MgSO_4$), filtered and concentrated. Purification using flash column chromatography on silica gel (petroleum ether/EtOAc, 95:5) yielded **3** (7.22 g, 67%) as a colorless oil: $[\alpha]^{25}_D$ 56.61 (*c* 1.09, $CH_2Cl_2$); IR (neat) 2931, 1699, 1489, 1428, 1364, 1243, 1168, 1108, 867, 733, 823, 738, 701 614, 503 cm$^{-1}$; $^1$H NMR (400 MHz, $CDCl_3$) δ 7.62–7.58 (m, 4H), 7.45–7.35 (m, 6H), 6.52 (dd, $J$ = 17.4, 10.6 Hz, 1H), 6.34 (app d, $J$ = 17.3 Hz, 1H), 5.82 (app d, $J$ = 10.6 Hz, 1H), 5.59 (d, $J$ = 7.4 Hz, 1H), 4.68 (ddd, $J$ = 7.8, 4.0, 4.0 Hz, 1H), 4.01 (dd, $J$ = 10.4, 3.4 Hz, 1H), 3.94 (dd, $J$ = 10.4, 3.8 Hz, 1H), 1.46 (s, 9H), 1.01 (s, 9H); $^{13}$C NMR (100 MHz, $CDCl_3$ ) δ 196.8, 155.5, 135.8, 135.7, 133.3, 133.0, 132.9, 130.1, 129.7, 128.0, 79.9, 64.4, 59.6, 28.6, 27.0, 19.4; HRMS (ESI) calcd for $C_{26}H_{36}NO_4Si$ [M + H]$^+$ *m/z* 454.2408, found 454.2424.

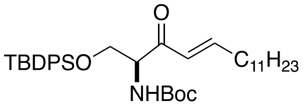

**6**

**(2*S*,4*E*)-1-(*tert*-Butyldiphenylsilyloxy)-2-(*N*-*tert*-butyloxycarbonylamino)hexadec-4-en-3-one (6).** Cat. **5** (30 mg, 0.04 mmol) was added to a solution of (2*S*)-1-(*tert*-butyldiphenylsilyloxy)-2-(*N*-tert-butyloxycarbonylamino)-4-pentene-3-one (**3**) (2.02 g, 0.441 mmol) and 1-tridecene (**4**) (2.2 mL, 8.8 mmol) in dry $CH_2Cl_2$ (65 mL). The reaction was allowed to stir in a preheated oil bath at 40 °C for 4h. The solvent was evaporated, and the crude product was purified using flash column chromatography on silica gel (petroleum ether/EtOAc, 95:5) to give **6** (2.38 g, 88%) as a slightly brownish oil: $[\alpha]^{25}_D$ 41.8 (*c* 1.0, $CH_2Cl_2$); $^1$H NMR (400 MHz, $CDCl_3$) δ 7.62–7.58 (m, 4H), 7.44–7.34 (m, 6H), 6.96 (dt, $J$ = 14.1, 6.8 Hz, 1H), 6.23 (app d, $J$ = 15.7 Hz, 1H), 5.62 (d, $J$ = 7.7 Hz, 1H), 4.63 (ddd, $J$ = 7.9, 4.0, 4.0 Hz, 1H), 3.97 (dd, $J$ = 10.4, 3.6 Hz, 1H), 3.92 (dd, $J$ =

14.4, 4.0 Hz, 1H), 2.20 (dt, *J* = 7.0, 7.0 Hz, 2H), 1.45 (s, 9H), 1.31–1.26 (m, 19H), 1.01 (s, 9H), 0.88 (t, *J* = 6.7 Hz, 3H), 0.82 (s, 9H); $^{13}$C NMR (100 MHz, $CDCl_3$) δ 196.4, 155.5, 149.7, 135.8, 135.7, 133.1, 133.1, 130.0, 127.9, 127.9, 127.0, 79.8, 64.6, 59.6, 32.9, 32.1, 29.8, 29.8, 29.7, 29.6, 29.5, 29.4, 28.6, 28.2, 26.9, 22.9, 19.5, 14.4; HRMS (ESI) calcd for $C_{37}H_{58}NO_4Si$ $[M + H]^+$ *m/z* 608.4130, found 608.4124.

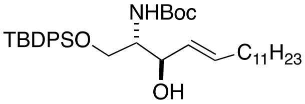


**7**

**(2*S*,3*R*,4*E*)-1-(*tert*-Butyldiphenylsilyloxy)-2-(*N*-*tert*-butyloxycarbonylamino) hexadec-4-en-3-ol (7).** $LiAl(Ot\text{-}Bu)_3H$ (5.03 g, 19.8 mmol) was added to (2*S*,4*E*)-1-(*tert*-butyldiphenylsilyloxy)-2-(*N*-*tert*-butyloxycarbonylamino)hexadec-4-ene-3-one (**6**) (5.48 g, 8.99 mmol) in dry EtOH (36 mL) at –78 °C. After being stirred for 5 h, aqueous HCl (1M, 20 mL) was added slowly. The mixture was then diluted with $H_2O$ (50 mL). The reaction mixtures was then extracted with EtOAc (3 X 50 mL). The combined organic extracts were dried ($MgSO_4$), filtered and concentrated. Purification by flash chromatography on silica gel (petroleum ether/EtOAc, 95:5 to 90:10) gave **7** (4.08 g, 74%) as a colorless oil: $[\alpha]^{25}_{D}$ 13.05 (*c* 1.64, $CH_2Cl_2$); IR (neat) 2924, 2854, 1698, 1498, 1428, 1365, 1169, 1111, 966, 822, 740, 701, 613, 504 $cm^{-1}$; $^{1}$H NMR (400 MHz, $CDCl_3$) δ 7.64 (t, *J* = 12.2 Hz, 4H), 7.46–7.36 (m, 6H), 5.78 (dt, *J* = 14.4, 6.5 Hz, 1H), 5.48 (dd, *J* = 15.4, 6.0 Hz, 1H), 5.19 (d, *J* = 7.0 Hz, 1H), 4.24 (app ddd, *J* = 6.5, 6.5, 6.5 Hz, 1H), 3.91 (dd, *J* = 10.5, 3.6 Hz, 1H), 3.76 (dd, *J* = 11.1, 3.1 Hz, 1H), 3.66 (br s, 1H), 3.15 (app d, *J* = 6.3 Hz, 1H), 2.04 (dt, *J* = 6.7, 6.7 Hz, 2H), 1.45 (s, 9H), 1.37–1.32 (m, 3H), 1.31–1.26 (m, 15H), 1.07 (s, 9H), 0.88 (t, *J* = 6.6 Hz, 3H); $^{13}$C NMR (100 MHz, $CDCl_3$ ) δ 156.1, 135.8, 133.6, 132.8, 132.8, 130.2, 129.4, 128.1, 79.7, 74.5, 64.3, 55.3, 32.6, 32.1, 29.9, 29.9, 29.8, 29.7, 29.6, 29.5, 29.4, 28.6, 27.1, 22.9, 19.4, 14.3; HRMS (ESI) calcd for $C_{37}H_{60}NO_4Si$ $[M + H]^+$ *m/z* 610.4286, found 610.4309.

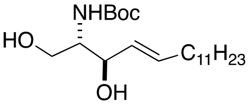


**8**

**(2*S*,3*R*,4*E*)-2-(*N*-*tert*-Butyloxycarbonylamino)hexadec-4-en-1,3-diol (8).**

TBAF (1.0 M in THF, 6.6 mmol, 6.6 mL) was added drop-wise to a solution of (2*S*,3*R*,4*E*)-1-(*tert*-butyldiphenylsilyloxy)-2-(*N*-*tert*-butyloxycarbonylamino)hexadec-4-en-3-ol (**7**) (1.82 g, 2.98 mmol) in THF (60 mL) at 0 °C. The cooling bath was removed, and the mixture was stirred for 2 h at rt. Saturated aqueous $NH_4Cl$ (30 mL) was added. The solution was extracted with $Et_2O$ (3 X 100 mL). The combined organic extracts

were dried ($MgSO_4$), filtered and concentrated. Purification by flash chromatography on silica gel (petroleum ether/EtOAc, 60:40) provided **8** (0.82 g, 74%) as a white solid: mp 54.1–55.4 °C; $[\alpha]^{25}_{D}$ –2.57 (*c* 1, $CH_2Cl_2$); IR (neat) 3342, 2916, 2849, 1687, 1531, 1464, 1365, 1244, 1172, 1062, 1004, 906, 649 $cm^{-1}$; $^1H$ NMR (400 MHz, $CDCl_3$) δ 5.75 (dt, *J* = 14.6, 6.6 Hz, 1H), 5.50 (dd, *J* = 15.4, 6.3 Hz, 1H), 5.34 (d, *J* = 8.0 Hz, 1H), 4.27 (app ddd, *J* = 5.0, 5.0 Hz, 1H), 3.90 (dd, *J* = 11.2, 3.2 Hz, 1H), 3.67 (app d, *J* = 11.0 Hz, 1H), 3.57 (br s, 1H), 3.10 (br s, 2H), 2.03 (dt *J* = 6.5, 6.5 Hz, 2H), 1.43 (s, 9H), 1.35 (t, *J* = 14.9 Hz, 2H), 1.24 (s, 16H), 0.86 (t, *J* = 6.0 Hz, 3H); $^{13}C$ NMR (100 MHz, $CDCl_3$) δ 156.5, 134.2, 129.1, 80.0, 74.8, 65.3, 55.7, 32.5, 32.1, 29.8, 29.8, 29.7, 29.5, 29.4, 29.3, 28.6, 22.9, 14.3; HRMS (ESI) calcd for $C_{21}H_{42}NO_4$ $[M + H]^+$ *m/z* 372.3108, found 372.3133.

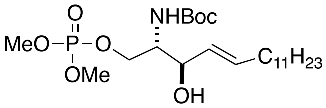

**9**

**(2*S*,3*R*,4*E*)-2-(*N-tert*-butyloxycarbonylamino)hexadec-4-en-3-ol-1-methyl phosphate (9).** $P(OMe)_3$ (0.04 mL, 0.034 mmol) was added drop-wise to a solution of (2*S*,3*R*,4*E*)-2-(*N-tert*-butyloxycarbonylamino)hexadec-4-en-1,3-diol (**8**) (100 mg, 0.27 mmol) and $CBr_4$ (0.123 g, 0.371 mmol) in pyridine (0.13 mL) at 0 °C. The reaction was stirred at the same temperature for 4.5 h, after which the precipitate formed was filtered off and rinsed with EtOAc (15 mL). The filtrate was washed with brine (10mL). The two layers were separated and the organic layer was dried ($MgSO_4$), filtered and concentrated. The crude product was purified using flash column chromatography on silica gel (petroleum ether/EtOAc, 60:40) to yield **9** (41 mg, 32%) as a colorless oil: $[\alpha]^{25}_{D}$ 5.56 (*c* 0.17, $CH_2Cl_2$); IR (neat) 2923, 2853, 1714, 1457, 1365, 1249, 1171, 1036, 825 $cm^{-1}$; $^1H$ NMR (400 MHz, $CDCl_3$) δ 5.76 (dt, *J* = 14.9, 6.7 Hz, 1H), 5.48 (dd, *J* = 15.4, 6.7 Hz, 1H), 5.06 (d, *J* = 7.0 Hz, 1H), 4.31 (dddd, *J* = 6.0, 6.0, 6.0, 6.0 Hz, 1H), 4.13–4.08 (m, 2H), 3.77 (d, *J* = 10.7 Hz, 6H), 3.04 (br s, 1H), 2.01 (dt *J* = 6.8, 6.8 Hz, 2H), 1.42 (s, 9H), 1.37–1.33 (m, 2H), 1.30–1.24 (m, 17H), 0.86 (t, *J* = 6.5 Hz, 3H); $^{13}C$ NMR (100 MHz, $CDCl_3$) δ 134.9, 128.7, 79.9, 72.6, 66.9 (d, $J_{C\text{-}P}$ = 2.8 Hz), 55.1, 54.7 (d, $J_{C\text{-}P}$ = 5.5 Hz), 32.5, 32.1, 29.9, 29.8, 29.8, 29.7, 29.5, 29.4, 29.3, 28.5, 22.9, 14.3; $^{31}P$ NMR (162 MHz, $CDCl_3$) δ 1.85; HRMS (ESI) calcd for $C_{23}H_{47}NO_7P$ $[M + H]^+$ *m/z* 480.3085, found 480.3092.

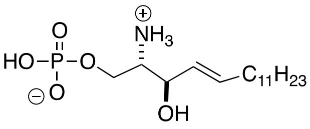

**S1P d16:1**

**(2*S*,3*R*,4*E*)-2-Ammoniumhexadec-4-en-3-ol-1-phosphate (S1P d16:1).** TMSBr (0.09 mL, 0.70 mmol) was added drop-wise to a solution of (2*S*,3*R*,4*E*)-2-aminohexadec-4-

en-3-ol-1-methyl phosphate (**9**) (0.04 g, 0.08 mmol) in $CH_2Cl_2$ (0.43 mL) at 0 °C. The reaction was stirred at rt for 30 min. AcOH (0.5 mL) was added, followed by the addition of ice water until precipitates were formed. The mixture was centrifuged and the $H_2O$ layer was removed. The solid residue was further washed with $H_2O$ (3 X 10 mL) and centrifugation was repeated each time to remove the $H_2O$. The solid residue was washed with a mixture of acetone/$H_2O$ (1:1 v/v, 3 X 5 mL). The sample was then left on the high vacuum to remove excess solvents to provide **S1P d16:1** (19 mg, 63%) as a white solid: mp 154.9–156.4 °C; $[\alpha]^{25}_{D}$ 4.15 (*c* 0.17, MeOH); IR (neat) 3435, 2918, 2849, 1543, 1463, 1243, 1028, 923, 510 $cm^{-1}$; $^1H$ NMR (400 MHz, MeOD) δ 5.90 (dt, *J* = 14.7, 6.8 Hz, 1H), 5.52 (dd, *J* = 15.4, 7.0 Hz, 1H), 4.30 (dd, *J* = 5.9, 5.9 Hz, 1H), 4.12 (ddd, *J* = 11.6, 7.3, 3.4 Hz, 1H), 4.04–3.96 (m, 1H), 3.39–3.33 (m, 1H), 2.13 (dt *J* = 7.0, 7.0 Hz, 2H), 1.46 (quin, *J* = 7.2 Hz, 2H), 1.39–1.32 (m, 17H), 0.93 (t, *J* = 7.0 Hz, 3H); $^{13}C$ NMR (100 MHz, MeOD) δ 135.7, 127.0, 63.6, 61.8 (d, $J_{C\text{-}P}$ = 4.8 Hz), 56.4 (d, $J_{C\text{-}P}$ = 6.4 Hz), 32.2, 31.9, 29.6, 29.5, 29.6, 29.5, 29.3, 29.2, 29.0, 22.5, 13.2, –1.2; $^{31}P$ NMR (162 MHz, MeOD) δ 1.31; HRMS (ESI) calcd for $C_{16}H_{35}NO_5P$ $[M + H]^+$ *m/z* 352.2247, found 352.2286.

Reference:

1. "Enantiomerically Pure α-Amino Acid Synthesis via Hydroboration−Suzuki Cross-Coupling," Collier, P. N.; Campbell, A. D.; Patel, I.; Raynham, T. M.; Taylor, R. J. K. *J. Org. Chem*. **2002**, *67*, 1802–1815.
